# Supplementary material for: Spatial Variation in Stream-Water Bacterial Communities Across Sites Differing in Wild Chinese Giant Salamander Detection Frequency
Source: Biology (Basel). 2026 Sep 18;15(18):1647. doi: 10.3390/biology15181647 (PMC13604818; doi:10.3390/biology15181647)
Supplement: Supplementary file 1 [file biology-15-01647-s001.zip › Data Related to “Spatial Variation in Stream-Water Bacterial Communities across Sites Differing in Wild Chinese Giant Salamander Detection Frequency”/Microbial Data/report/src/hyperlinks/Methods_QIIME2_EN_LRS_hw.pdf]

Methods

1 Experimental Procedure

1.1 Genomic DNA Extraction and Quality Assessment

Genomic DNA was extracted according to sample type. The extracted DNA was then subjected to quality assessment via 1% agarose gel electrophoresis (120V, 30 min) to evaluate band integrity and nucleic acid purity. Qualified DNA samples were diluted with sterile nuclease-free water to a final concentration of 1
ng/μL for downstream experiments.

1.2 PCR Amplification and Product Purification

The diluted genomic DNA was used as the template for PCR amplification of the target 16S rRNA gene region using barcode-tagged primers. PCR was performed in a reaction containing 12.5 μL KAPA HiFi HotStart ReadyMix, 2.5 μM primers, and 10 ng genomic DNA template. The thermal cycling conditions were as follows: initial denaturation at 95℃ for 3 min, followed by 22 cycles of denaturation at 98℃ for 20 s, annealing at 57℃ for 30 s, and extension at 72℃ for 75 s, with a final extension at 72℃ for 5 min. The PCR products were verified by 2% agarose gel electrophoresis and purified using magnetic bead-based purification kits (e.g., AMPure XP) to remove primer dimers.

1.3 Kinnex Library Preparation

Barcoded PCR products from different samples were pooled in equimolar amounts, and the Kinnex long-read sequencing technology was employed for library preparation. The detailed steps are as follows:

End Repair: PCR products were treated with Kinnex End Repair Enzyme (20°C, 30 min) to ensure blunt-ended DNA fragments.

Adapter Ligation: Kinnex-specific sequencing adapters (20 μM) and T4 DNA ligase (20°C, 15 min) were added to ligate DNA fragments to sequencing adapters.

Linear Array Assembly: The 16S rRNA gene fragments were concatenated into long linear DNA arrays using Kinnex Assembly Enzyme (37°C, 1 h).

Library Purification: SMRTbell® magnetic beads were used for size selection (0.45× and 0.8× bead ratios) to retain target fragments ranging from 500 to 2000 bp, thereby improving library quality.

1.4 Library Quality Evaluation and Sequencing

The purified library was quantified using a Qubit® 4.0 Fluorometer, and its size distribution

was assessed using an Agilent 2100 Bioanalyzer to ensure the primary peak fell within the

expected range (1.5–3 kb). Libraries meeting quality standards were sequenced on the Revio platform using Single Molecule, Real-Time (SMRT) sequencing to obtain high-quality long-read data.

2 Bioinformatics Analysis Pipeline

2.1 Long Reads Quality Control

Quality filtering on the raw tags were performed using the dada2 software to obtain high-quality Clean Tags.

2.2 ASVs Denoise and Species Annotation

2.2.1 ASVs Denoise

For the Effective Tags obtained previously, denoise was performed with DADA2 or deblur module in the QIIME2 software to obtain initial ASVs (Amplicon Sequence Variants) (default: DADA2) .

2.2.2 Species Annotation

Species annotation was performed using QIIME2 software. For 16S/18S, the annotation database is Silva Database, while for ITS, it is Unite Database.

2.2.3 Phylogenetic Relationship Construction

In order to study phylogenetic relationship of each ASV and the differences of the dominant species among different samples(groups), multiple sequence alignment was performed using QIIME2 software.

2.2.4 Data Normalization

The absolute abundance of ASVs was normalized using a standard of sequence number corresponding to the sample with the least sequences. Subsequent analysis of alpha diversity and beta diversity were all performed based on the output normalized data .

2.2.5 Relative abundance

Top 10 taxa of each samples at each taxonomic ranks(Phylum, Class, Order, Family, Genus, Species) were selected to plot the distribution histogram of relative abundance in Perl through SVG function.

2.2.6 Heatmap

The abundance information of top 35 taxa of each samples at each taxonomic ranks were used to drawn the heatmap, which visually display different abundance and taxa clustering. This was achieved in R through the pheatmap() function.

2.2.7 Ternary plot

Ternary plot for top 10 taxa at each taxonomic ranks can be used to show the abundance difference among three samples. It was performed in R with vcd() function.

2.2.8 Venn and Flower diagram

Venn and Flower diagrams visually display the common and unique information between different samples or groups. Venn and Flower diagrams were produced in R with VennDiagram() function and in perl with SVG function, respectively.

2.2.9 Phylogenetic tree

Phylogenetic tree, also called evolutionary tree, can describe the evolutionary relationship between different species. One hundred genera with the highest abundance in the samples were selected and performed sequence alignment to draw the phylogenetic tree in perl with SVG function.

2.3 Alpha Diversity

2.3.1 Alpha Diversity

In order to analyze the diversity, richness and uniformity of the communities in the sample, alpha diversity was calculated from 7 indices in QIIME2, including Observed_otus, Chao1, Shannon, Simpson, Dominance, Good's coverage and Pielou_e.

Three indices were selected to identify community richness:

Observed_otus – the number of observed species (http://scikit-bio.org/docs/latest/generated/skbio.diversity.alpha.observed_otus.html);

Chao – the Chao1 estimator (http://scikit-bio.org/docs/latest/generated/skbio.diversity.alpha.chao1.html);

Dominance – the Dominance index (http://scikit-bio.org/docs/latest/generated/skbio.diversity.alpha.dominance.html);

Two indices were used to identify community diversity:

Shannon – the Shannon index (http://scikit-bio.org/docs/latest/generated/skbio.diversity.alpha.shannon.html);

Simpson – the Simpson index (http://scikit-bio.org/docs/latest/generated/skbio.diversity.alpha.simpson.html);

One indice was used to calculate sequencing depth:

Coverage – the Good's coverage (http://scikit-bio.org/docs/latest/generated/skbio.diversity.alpha.goods_coverage.html);

One indice was used to calculate species evenness:

Pielou_e – Pielou's evenness index (http://scikit-bio.org/docs/latest/generated/skbio.diversity.alpha.pielou_e.html).

2.3.2 Species accumulation boxplot

In order to evaluate the richness of microbial community and sample size. Species accumulation boxplot can be used to visualize, which performed with vegan package in R software.

2.4 Beta Diversity

2.4.1 Beta diversity

In order to evaluate the complexity of the community composition and compare the differences between samples(groups), beta diversity was calculated based on weighted and unweighted unifrac distances in QIIME2.

2.4.2 Beta diversity heatmap

Beta diversity analysis was used to evaluate differences of samples in species complexity. Beta diversity on both weighted and unweighted unifrac were calculated by QIIME2 software. Then a heatmap was created to display the unifrac distance between samples, which was realized in Perl.

2.4.3 Unweighted Pair-group Method with Arithmetic Mean (UPGMA)

A cluster tree is constructed on UPGMA, which is based on the weighted unifrac distance matrix. This is widely used in ecology for evolutionary classification. UPGMA diagram was drawn through the upgma.tre function within Qiime2.

2.4.4 Cluster analysis

Cluster analysis was preceded by principal component analysis (PCA), which was applied

to reduce the dimension of the original variables using the ade4 package and ggplot2 package with R software(Version 4.0.3).

Principal Coordinate Analysis (PCoA) was performed to get principal coordinates and visualize from complex and multidimensional data. A distance matrix of weighted or unweighted unifrac among samples was obtained before transformation to a new set of orthogonal axes, by which the maximum variation factor is demonstrated by first principal coordinate, and the second maximum one by the second principal coordinate, and so on. PCoA analysis was displayed by ade4 package and ggplot2 package in R software(Version 4.0.3).

Non-metric multidimensional scaling (NMDS) was also implemented for data dimension reduction. Similar to the PCoA, NMDS also use the distance matrix but it emphasizes the numerical rank instead. The distance between sample points on the diagram can only reflect the rank information rather than the numerical differences. NMDS analysis was implemented through R software with ade4 package and ggplot2 package.

2.5 Community difference analysis

A series of statistical analyses which inlcude Anosim, Adonis, Multi-response permutation procedure (MRPP), Simper, T-test, MetaStat and LEfSe, were performed to reveal the community structure differentiation.

Anosim, Adonis and MRPP analysis are non-parametric tests that analyze the difference between high-dimensional data group. They can test whether the differences between groups are significant greater than the differences within the group, which can determine whether the grouping is meaningful. All of them were performed with vegan package and ggplot2 package within R.

The Wilcoxon rank-sum test (also known as the Mann–Whitney U test) is a nonparametric statistical method for comparing two independent samples. The null hypothesis states that the two populations from which the samples are drawn exhibit identical distributions. By analyzing the average ranks of the samples, this test determines whether the distributions differ significantly. It can be applied to assess differences in species composition between groups.

The Kruskal-Wallis H test (Kruskal-Wallis rank-sum test) is a nonparametric method that extends the two-sample Wilcoxon rank-sum test to multiple independent samples (⩾3 groups). This analysis can assess significant differences in species composition across multiple sample groups.

Simper can reveal the contribution of each species to the differentiation between groups. Top 10 species were selected and presented on the graph. It was performed in R with Vegan package and ggplot2 package.

MetaStat can showcase the species that display significant differences between groups. It is based on the multiple hypothesis test and performed in R with ComplexHeatmap package.

LEfSe is widely used to discover biomarkers and it can reveal metagenomic characteristics. To achieve this, a exclusive package named lefse was utilized.

2.6 Function prediction

PICRUSt (V1.1.4) is a package in R and is mainly used to predict the metagenomic functions based on marker genes. PICRUSt2 (V2.3.0) is the improved version of PICRUSt.

Tax4Fun (V0.3.1) is a R package that is widely used for intestinal and soil samples. In general, it can supply more accurate results when compared with PICRUSt, especially for soil samples.

BugBase is an excellent tool to discover the phenyotpe of microogranisms. It can classify the microbial communities according to seven phenotypes: Gram Positive, Gram Negative, Biofilm Forming, Pathogenic, Mobile Element Containing, Oxygen Utilization, including Aerobic, Anaerobic, and Cultivable Anaerobic, and Oxidative Stress Tolerance.

FunGuild is an excellent tool through python when working with fungi samples.

FAPROTAX through python can play a great role when elucidating the possible biochemical processes and elements in play.

2.7 Association analysis

To explore the symbiotic relationship between species and to reveal the environmental factor influence on community structures, 2D and 3D network diagrams were drew for visualization. Further analyses such as spearman correlation test, canonical correspondence analysis (CCA)/redundancy analysis (RDA) and dbRDA can be used to reflect the correlation between

environmental factors and species abundance. All of these diagram and analysis were completed in R.

2.8 Community Assembly Mechanisms

To investigate the influence of environmental factors on microbial community assembly processes, we employed the Beta Nearest Taxon Index (βNTI) analysis. βNTI is a phylogenetic β-diversity metric that quantifies the degree of divergence between the most phylogenetically related taxa (nearest taxon) across samples. Specifically, βNTI compares the observed phylogenetic turnover (βMNTD, beta mean nearest taxon distance) to null model expectations, thereby distinguishing whether community differences are primarily driven by stochastic processes or deterministic selection.

2.9 Python, R and Perl

Throughout the whole analysis, the version of Python, R and Perl used are 3.6.13, 4.0.3 and 5.26.2, respectively.

3 Reference

Bokulich NA, Subramanian S, Faith JJ, et al. Quality- filtering vastly improves diversity estimates from Illumina amplicon sequencing.Nature Methods . 2012; 10(1):57-59. doi:10. 1038/nmeth.2276.

Edgar RC, Haas BJ, Clemente JC, Quince C, Knight R. UCHIME improves sensitivity and speed of chimera detection.Bioinformatics. 2011;27(16):2194-2200. doi:10. 1093/bioinformatics/btr381.

Magoc T, Salzberg SL. FLASH: fast length adjustment of short reads to improve genome assemblies.Bioinformatics. 2011;27(21):2957-2963. doi:10. 1093/bioinformatics/btr507.

Wang Y, Guo H, Gao X, Wang J. The Intratumor Microbiota Signatures Associate With Subtype, Tumor Stage, and Survival Status of Esophageal Carcinoma. Frontiers in Oncology . 2021; 1 1. doi:10.3389/fonc.2021.754788.
